# Characterizing superficial epidermolytic ichthyosis in a patient with KRT2 mutation responsive to ustekinumab

**DOI:** 10.1016/j.jdcr.2026.01.028

**Published:** 2026-01-27

**Authors:** Mallory Zaino, Jordan Jones, Ronghua Hu, Keith Choate, Jeffrey P. Callen, Courtney R. Schadt

**Affiliations:** a Division of Dermatology, University of Louisville School of Medicine, Louisville, Kentucky; b Department of Dermatology, Yale University School of Medicine, New Haven, Connecticut; c Department of Genetics, Yale University School of Medicine, New Haven, Connecticut; d Department of Pathology, Yale University School of Medicine, New Haven, Connecticut

**Keywords:** biologic, epidermolytic, ichthyosis, IL-12/23, IL-17, keratinopathic, NG

The ichthyoses encompass a heterogeneous group of genetic epidermal differentiation disorders characterized by varying degrees of erythema, scale, and xerosis.^1^^,^^2^ Nonsyndromic keratinopathic ichthyoses associated with keratin gene mutations include KRT2-nEDD, KRT1/10-nEDD-epidermolytic, and KRT1/10-nEDD-revertant mosaic, previously known as superficial epidermolytic ichthyosis (SEI), epidermolytic ichthyosis, and ichthyosis with confetti, respectively.^3^ Treatment is directed toward managing barrier dysfunction and reducing scaling but may be a challenge.

Here, we present a 28-year-old African American female referred to dermatology for erythematous annular, nearly circinate, scaly patches and plaques, some with lateral vesiculation, on the trunk and extremities (Figs 1 and 2). Corrugated scale in the antecubital fossae was also noted. Punch biopsy taken from a plaque revealed psoriasiform epidermal changes and epidermolytic hyperkeratosis (Fig 3). Direct immunofluorescence studies were negative. She had previously failed dupilumab, methotrexate, and cyclosporine and was initiated on ustekinumab 45 mg subcutaneous at weeks 0 and 4, then every 12 weeks. Within 12 weeks of starting ustekinumab, she showed significant improvement (Fig 4).

**Fig 1:**
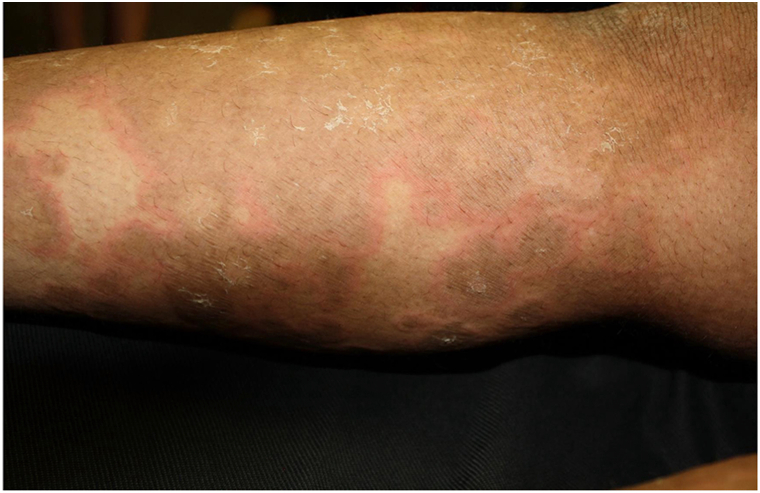
Annular scaly hypopigmented patches with a peripheral rim of erythema on the right leg.

**Fig 2:**
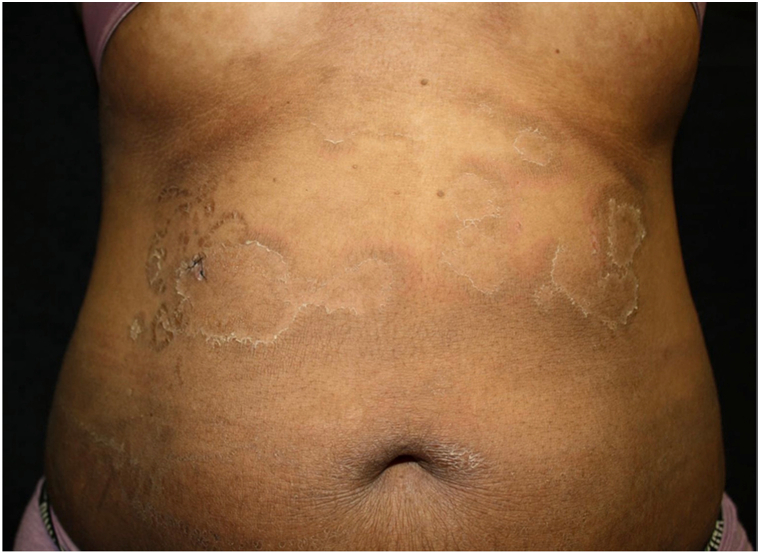
Annular scaly hypopigmented patches with hyperpigmented accentuated skin lines on the trunk.

**Fig 3:**
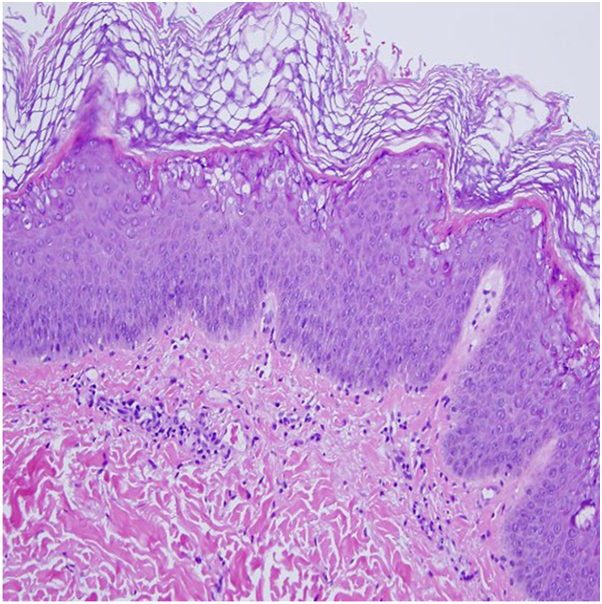
Psoriasiform epidermal change with epidermolytic hyperkeratosis.

**Fig 4:**
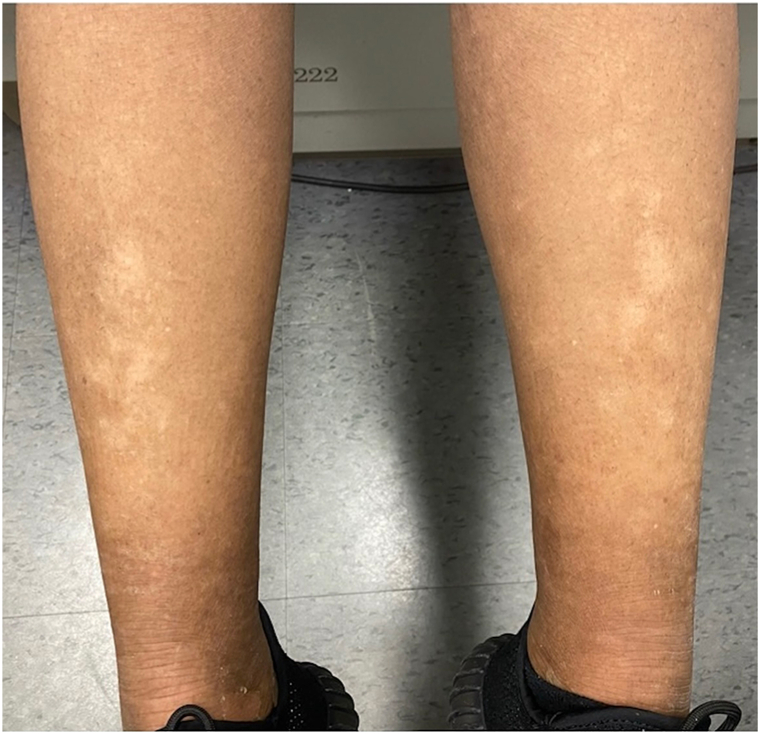
Subtle hypopigmented patches on the shins.

Multiple family members of the patient had similar undiagnosed skin findings. This motivated genetic evaluation of the index case and a cross-sectional study assessing clinical characteristics of other affected relatives. Eight (5 males and 3 females) family members spanning 3 generations demonstrated clinical findings consistent with ichthyosis. Two (25%) of the 8 had clinicopathologically confirmed diagnosis. Median (range) age of participants at the time of study was 23 (1-63) years.

Median (range) age of disease onset was 8 (2-60) months. Median (range) Visual Index for Ichthyosis Severity Scale (VIIS) was 5.5 (2-9). Median (range) VIIS for individuals in the first, second, and third generation were 9 (0), 5 (2-6), and 7 (4-7), respectively. Additional anatomic areas were assessed for VIIS scores (Table I). Mean (SD) body surface area for scale and erythema was 30.03% (10.16%) and 5.22% (5.25%), respectively (*P* < .0001). Keratoderma was present with prominent corrugated hyperkeratosis at flexures, scattered plate-like scale on the lower legs, and areas of superficial erosion intermixed with bullae on the lower legs. Palmoplantar keratoderma was not observed. No patient had a history of involvement of the nails or mucous membranes, and most reported a relapsing-remitting course of disease. Next-generation sequencing identified a novel mutation in the KRT 2 gene (c. 1462G>A, p. Glu488Lys).

**Table I:** Visual Index of Ichthyosis Severity (VIIS) for scale and erythema

Anatomic location	Scale	Erythema
	Mean (SD)	Mean (SD)
Upper back	0.25 (0.46)	0 (0)
Upper arm	1.43 (1.19)	0 (0)
Lower leg	1.88 (1.13)	0.5 (0.76)
Dorsal foot	1.63 (0.74)	0 (0)
Axilla	0.5 (0.76)	0.13 (0.35)
Lower back	1.25 (0.89)	0 (0)
Abdomen	1.13 (0.64)	0.63 (0.74)
Angle	2.63 (0.92)	0.25 (0.71)

This case series demonstrates the phenotypic variability that may occur in SEI and is the first study to date of an SEI patient with a *KRT2* mutation responsive to ustekinumab. The significance of the identified missense mutation resulting in a glutamate > lysine substitution at position 488 in the context of treatment response to ustekinumab is not known.

Inhibition of the Th17/IL-23 pathway may reduce levels of inflammatory cytokines associated with abnormal cornification, lipid homeostasis, and keratinocyte proliferation associated with disease phenotype.^3^, ^4^, ^5^ Recently, a T helper (Th)17 cell dominant immune profile of patients with ichthyoses has supported a role for targeted therapeutics used in psoriasis, though broad efficacy has not been demonstrated, and a trial of secukinumab showed inconsistent, variable response across genotypes.^3^ There is 1 ongoing clinical trial (NCT04549792) on the efficacy and safety of ustekinumab treatment in patients with ichthyoses and various reports in the literature of ichthyosiform erythrodermic patients responding to ustekinumab treatment.^6^, ^7^, ^8^, ^9^, ^10^ Additional studies on the use of biologics in patients with genetic disorders of keratinization are likely needed.

This study is limited by age-dependent phenotypic variability, which may affect VIIS and body surface area scores across generations. Clinical severity scores for patients of skin of color are scarce, but the VIIS has been validated across Fitzpatrick skin types I-VI and may be used to assess keratinopathic ichthyoses patient response to biologics as continued investigations take place.^11^^,^^12^

## Appendix 1 methods for NGS

Genomic DNA was isolated using a standard phenol-chloroform protocol. Bar-coded DNA libraries were prepared and exome capture was performed by the Yale Center for Genome Analysis. Illumina Novaseq instruments were used for sequencing samples with 75 bp paired-end reads. Resulting reads were aligned to the human reference genome (hg19), sequence was trimmed to targeted intervals, PCR duplicates were removed, and single nucleotide and indel variants were identified using SAMtools software. Variants were annotated for functional impact and filtered to examine coding mutations with quality scores ≥50 and to exclude frequent variants present in dbSNP, ExAC and gnomAD. Aligned reads were examined with the Broad Institute Integrative Genomics Viewer (IGV). Sanger sequencing verification of mutations and sequencing of parental DNA was performed via PCR using Kapa 2G Fast polymerase (Kapa Bio systems) and Sanger sequencing. Primers were designed with ExonPrimer and SNPmasker.

## Conflicts of interest

Dr Choate has served as an investigator for AbbVie, Anaptyss, Janssen, Mayne, and Regeneron. Dr Callen has received honoraria for adjudication for Serono, Biogen, and Riovant. Drs Zaino, Jones, Ranghua, Boyden, and Schadt have no conflicts to disclose.
